# A hybrid machine learning-based model for predicting flight delay through aviation big data

**DOI:** 10.1038/s41598-024-55217-z

**Published:** 2024-02-26

**Authors:** Min Dai

**Affiliations:** CAAC Academy, Civil Aviation Flight University of China, Guanghan, 618307 China

**Keywords:** Machine learning, Big data, Aviation data, Flight delay prediction, Computational science, Computer science, Information technology

## Abstract

The prediction of flight delays is one of the important and challenging issues in the field of scheduling and planning flights by airports and airlines. Therefore, in recent years, we have witnessed various methods to solve this problem using machine learning techniques. In this article, a new method is proposed to address these issues. In the proposed method, a group of potential indicators related to flight delay is introduced, and a combination of ANOVA and the Forward Sequential Feature Selection (FSFS) algorithm is used to determine the most influential indicators on flight delays. To overcome the challenges related to large flight data volumes, a clustering strategy based on the DBSCAN algorithm is employed. In this approach, samples are clustered into similar groups, and a separate learning model is used to predict flight delays for each group. This strategy allows the problem to be decomposed into smaller sub-problems, leading to improved prediction system performance in terms of accuracy (by 2.49%) and processing speed (by 39.17%). The learning model used in each cluster is a novel structure based on a random forest, where each tree component is optimized and weighted using the Coyote Optimization Algorithm (COA). Optimizing the structure of each tree component and assigning weighted values to them results in a minimum 5.3% increase in accuracy compared to the conventional random forest model. The performance of the proposed method in predicting flight delays is tested and compared with previous research. The findings demonstrate that the proposed approach achieves an average accuracy of 97.2% which indicates a 4.7% improvement compared to previous efforts.

## Introduction

Prior knowledge of flight delays is a key prerequisite for the management and scheduling of flights in airports and airlines^1^. Flight delays can result from adverse weather conditions, aircraft malfunctions, unavailability of flight conditions, or even delays in previous flights^2^. Therefore, predicting flight delays is a highly complex process that can be influenced by multiple factors^3^. On the other hand, providing accurate predictions of future flight delays is of great importance to airports. By being aware of flight delays, airports and airline managers can take necessary measures to minimize the losses caused by delays and increase the efficiency of the aviation system^4^. Moreover, having a reliable flight delay prediction system can directly impact passenger satisfaction and increase airline revenue^5^. The significance of this issue has led to the development of various methods for predicting flight delays in previous research studies.

In most previous studies, machine learning techniques have been used for flight delay prediction. Initial research utilized classical machine learning techniques, while in recent years, researchers have focused more on employing ensemble learning models and deep learning^6^. However, the reported results in these studies indicate a noticeable gap with an ideal prediction system^1^. To reduce this gap, the problem of flight delay prediction needs to be examined from three aspects. Firstly, the prediction of flight delays should be based on relevant, accurate, and describable factors in an appropriate manner while avoiding the use of unrelated factors that can hinder the prediction process. As mentioned, the existence of a delay in a flight can result from various factors such as weather conditions, the flight’s delay history, congestion at departure and destination airports, aircraft characteristics, and the flight route. Some studies have only considered a limited number of these characteristics, while other models proposed in different studies include a large set of features that can potentially affect the prediction speed and accuracy. Therefore, determining the most relevant factors associated with flight delays is of great importance and requires a review of previous research in this area.

The second aspect to consider in the flight delay prediction problem is the massive volume of flight data, which requires the use of big data processing techniques. This feature has often made previously proposed models unable to be used in real-world applications. To address this issue, two strategies can be adopted. The first strategy involves utilizing distributed computing techniques, which have not been widely embraced in previous studies due to the complexity of the resulting models. The second strategy is simplifying the problem by breaking it down into several problems with lower complexity using big data processing techniques, which will be investigated in this study. By employing this strategy, it is possible to achieve a fast and flexible model for flight delay prediction that efficiently handles the processing of large flight data volumes.

The third aspect that needs to be considered in the flight delay prediction problem is the configuration of the learning model. In most previous studies, the configuration of the learning models has not received much attention. Consequently, it is difficult to ensure satisfactory performance of these strategies. In this study, it was attempted to guarantee higher performance of machine learning models by utilizing optimization techniques. In summary, the contributions of this paper are as follows:

- This paper proposes a clustering-based model for decomposing the flight delay prediction task into subproblems, improving the prediction system’s performance in terms of accuracy and speed when using large flight data. In this approach, initial samples are clustered using the DBSCAN algorithm, and a separate prediction model is utilized to analyze the samples in each cluster. This process significantly aids in pattern learning for the prediction models.
- This paper presents a novel weighted random forest model called COWRF (COA-optimized Weighted Random Forest) for flight delay prediction. COWRF is essentially a random forest model where each tree component is tuned using COA. The tuning processes occur at the split point of decision nodes and the weighting of each tree. Modifying the values of split points in each tree component in COWRF enhances its local accuracy, while the weighting strategy of tree components contributes to the overall accuracy improvement of the COWRF model.
- In this study, the importance and impact of each indicator on flight delays are examined using analysis of variance (ANOVA), and the most relevant indicators are identified using feature selection strategies. It is demonstrated that employing these indicators can effectively improve prediction accuracy.

It should be noted that the introduced COWRF model in this paper, as well as its distribution in data clusters for flight delay prediction, have innovative aspects that distinguish the current research from previous similar efforts. The continuation of this paper is organized as follows. The second section provides a review of previous research conducted in this field. The third section describes the proposed method for flight delay prediction based on big data and machine learning techniques. The fourth section presents the results and discusses the findings of the research, while the fifth section summarizes the research findings.

## Literature review

The importance of the flight delay problem has led to its investigation in numerous studies. Among them, a significant number of previous studies have attempted to solve this problem using machine learning techniques. For example, in Ref.^7^, a method based on spatio-temporal analysis was proposed for flight delay prediction. In this approach, spatial features of flights were extracted based on complex network theory. Additionally, by employing Long Short-term Memory (LSTM) models, the temporal correlation between weather conditions and airport traffic was modeled to predict these characteristics for each flight. Finally, a random forest model combining the temporal and spatial features was used to predict flight delays. The research was continued in Ref.^8^, where a Convolutional Neural Network (CNN) model was utilized to extract spatial features of flights, which proved to be more efficient in describing features compared to the complex graph theory-based model. Similar to Ref.^7^, in this study, the temporal features based on LSTM and spatial features based on CNN were merged to perform flight delay prediction using a random forest model.

In Ref.^9^, ensemble learning methods based on Gradient Boosting were used for flight delay prediction. This approach aimed to predict delays based on only seven flight-related features: airline type, aircraft type, departure airport, arrival airport, flight day, flight time, and distance. In this method, the mentioned features were preprocessed and encoded, and then three models, XGBoost, LightGBM, and CatBoost, were employed for delay prediction. This approach has two main shortcomings. Firstly, the considered number of features seems insufficient since providing accurate predictions requires a set of factors related to weather conditions and congestion. Secondly, each of the employed learning models requires tuning various hyperparameters to achieve satisfactory performance.

In Ref.^10^, a combination of machine learning models was used for flight delay prediction, aiming to address the limitations associated with Ref.^9^. In this approach, flight data was preprocessed, and then a combination of three models, XGBoost, LightGBM, and CatBoost, was employed for flight delay prediction. Bayesian techniques were utilized to tune the hyperparameters of each model. Additionally, the Synthetic Minority Oversampling Technique (SMOTE) was used to balance the number of samples with and without delays according to the schedule.

In Ref.^11^, a method for flight delay prediction through the analysis of direct and indirect indicators using machine learning techniques was proposed. In this research, a set of indicators such as weather conditions, airport congestion, flight routes, and flight characteristics (e.g., number of passengers, aircraft size, airline features, etc.) were introduced as direct indicators. Furthermore, factors such as the previous airport and previous flights were considered indirect indicators. Then, an LSTM model was used to predict the delays based on these indicators.

In Ref.^12^, a machine learning-based approach for strategic-level flight delay prediction was presented, distinguishing it from previous works. Strategic-level flight delay prediction covers a period of up to six months before the flight and the models based on it can serve as effective tools for flight schedule adjustments at airports and airline managers. In this approach, after preprocessing and feature selection, three models, Perceptron Neural Network, LightGBM, and Random Forest, were utilized for delay prediction.

The research conducted in Ref.^13^ utilized a Fully Connected Deep Neural Network (DFCNN) for flight delay prediction. In this method, the performance of different structures of the DFCNN model was analyzed and evaluated for delay prediction by examining weather information, flight characteristics, and historical flight delay data. Then, an optimized structure with the best performance was obtained by optimizing the parameters of the model from three aspects: activation function, input data, and delay threshold. The resulting DFCNN model consisted of five hidden layers with Exponential Linear Unit (ELU) activation functions and achieved an average prediction accuracy of 92.39%.

In Ref.^14^, a random forest model based on cluster computing techniques was used for flight delay prediction. The main focus of this research was to improve the processing speed of flight data using big data processing techniques, and significant improvement in prediction accuracy was not achieved. However, the random forest model was capable of predicting flight delays with an accuracy of 92.7%, indicating the compatibility of this learning model with the flight delay prediction problem. Consequently, the random forest has been used in many studies in this field. According to the reported results, the use of cluster computing techniques in the random forest model can lead to a 38% increase in processing speed.

In Ref.^15^, factors related to flight delays were divided into two categories: weather-dependent and weather-independent, and a probabilistic model based on a random forest was used to analyze the impact of each factor on prediction accuracy. Finally, a random forest was used to detect flight delays based on the selected factors.

The research conducted in Ref.^16^ proposed a Graph Convolutional Network (GCN)-based model called Geographical and Operational GCN (GOGCN) for flight delay prediction, which demonstrated better capability in representing geographical properties and spatio-temporal features compared to GCN alone. In this approach, global operational features and local geographical features were separately processed by two GCN models. Then, the extracted features from these two models were merged to perform flight delay prediction through an output layer.

In Ref.^17^, deep learning techniques were utilized for flight delay prediction. In this method, a set of flight-related and weather-related features were preprocessed, and the ECA-MobileNetV3 model was used to reduce the dimensionality of the features. Finally, the extracted features were classified using a SoftMax layer.

Research in Ref.^18^, combined severak deep learning models for flight delay prediction. This research initially employed a CNN-based model called CondenseNet, and by integrating CBAM components into this model, the prediction accuracy was improved. Additionally, a model combining CNN and LSTM was proposed for delay prediction, and by adding SimAM components to this CNN-LSTM model, it achieved a 91.36% prediction accuracy, showing a significant improvement compared to individual CNN, LSTM, and CondenseNet models. In Ref.^19^, a GCN-based model was presented for flight delay prediction, which represented temporal and spatial features in a separable manner and effectively learned the relationships between these features. In Ref.^20^, a model named Grasshopper Optimization Algorithm-based Random Forest (GHOA-RF) for predicting flight delay was presented. In this model, the GHOA was used for tuning the parameters of the RF model. Hybrid deep learning techniques are effective approaches for solving a wide range of problems such as traffic prediction^21,22^, trajectory prediction^23^, and so on. Also, optimization techniques have been used in many researches to improve the efficiency of learning models^24^. This research applies optimization techniques to machine learning models to achieve an accurate flight delay prediction system.

## Research method

To accurately predict flight delays, it is necessary to utilize relevant indicators and employ an optimized prediction model that can handle large-scale flight data processing. This section outlines the proposed method to meet these requirements.

### Database

Since existing databases for flight delay prediction are often limited, the required data for this research was collected using a web crawler from the FlightRadar24 website. In the data collection process, information for arriving flights at JFK International Airport, New York during June, July, and August 2023 was collected. The collected data includes information on 23,793 domestic and international flights to this airport. By excluding canceled flights (997 samples), the data was reduced to 22,796 records. In the obtained dataset, 18,591 samples belong to the category of on-time flights, while 4102 flights have minor delays, and 103 flights have significant delays.

Then, due to the imbalanced nature of samples in the target classes (low number of samples for flights with minimal and significant delays), information on delayed flights from January to May 2023 was also utilized. This operation, increased the instances count to 24,986, including 854 samples belonging to flights with significant delays and 5541 samples related to flights with small delays. For all samples in the database, information regarding origin and destination airport congestion, aircraft type, weather conditions, flight time, and airline delay history were collected. Table 1 presents the set of extracted features for describing each database sample.

**Table 1:** Set of extracted features for each database sample.

Category	ID	Title	Description	Data type
Weather condition	${I}_{1}$	Temperature	Temperature at the neighboring station of the origin airport (°C)	Continuous
	${I}_{2}$	Humidity percentage	Humidity at the neighboring station of the origin airport (%)	Continuous
	${I}_{3}$	Cloud density	Cloud density at the origin airport	Ordinal
	${I}_{4}$	Wind direction	Average wind direction at the station of the origin airport	Ordinal
	${I}_{5}$	Horizontal visibility	Horizontal visibility at the station of the origin airport (m)	Continuous
	${I}_{6}$	Wind speed	Average wind speed at the station of the origin airport (km/h)	Continuous
	${I}_{7}$	Air pressure	Air pressure at the station of the origin airport (bar)	Continuous
Flight status	${I}_{8}$	Day	Number of days elapsed in the month	Discrete
	${I}_{9}$	Month	Number of months elapsed in the year	Discrete
	${I}_{10}$	Day of the week	Number of days elapsed in the week	Discrete
	${I}_{11}$	Departure time	Scheduled departure time from the origin	Continuous
	${I}_{12}$	Arrival time	Scheduled arrival time at the destination	Continuous
	${I}_{13}$	Travel time	Predicted travel duration from takeoff to arrival at the destination (min)	Continuous
	${I}_{14}$	Flight distance	Flight distance between the origin and destination (km)	Continuous
	${I}_{15}$	Flight class	Airline flight class type	Ordinal
	${I}_{16}$	Flight type	Domestic (1) or international (2) flight	Ordinal
	${I}_{17}$	Aircraft	Aircraft type	Ordinal
	${I}_{18}$	Number of passengers	Total number of adult passengers on the flight	Continuous
	${I}_{19}$	Previous flight	Existence (1) or absence (2) of previous flight	Ordinal
	${I}_{20}{-}{I}_{26}$	Airline delay history	Delay history in the last 7 airline flights as a vector (min)	Discrete
Airport status	${I}_{27}$	Origin congestion level	Number of scheduled flights at the departure time from the origin airport	Discrete
	${I}_{28}$	Destination congestion level	Number of scheduled flights at the arrival time at the destination airport	Discrete
	${I}_{29}{-}{I}_{35}$	Airport delay history	Delay history in the last seven incoming flights at the airport as numerical values (min)	Discrete
	${I}_{36}$	Origin delay rate	Rate of delayed flights at the departure airport during the past 7 days	Continuous

According to Table 1, each record in the database is described through 36 different indicators. These indicators can be divided into three categories: weather conditions (7 indicators), flight status (19 indicators), and airport status (10 indicators).

### Ensemble learning-based flight delay prediction

The proposed method for flight delay prediction utilizes a combination of big data processing techniques, machine learning, and optimization. Additionally, statistical analysis of the indicators is used to determine the most relevant features related to flight delays. The proposed method includes three steps:

1. Preprocessing and selection of relevant indicators.
2. Clustering based on the DBSCAN algorithm.
3. Delay prediction based on the COWRF models.

**Figure 1:**
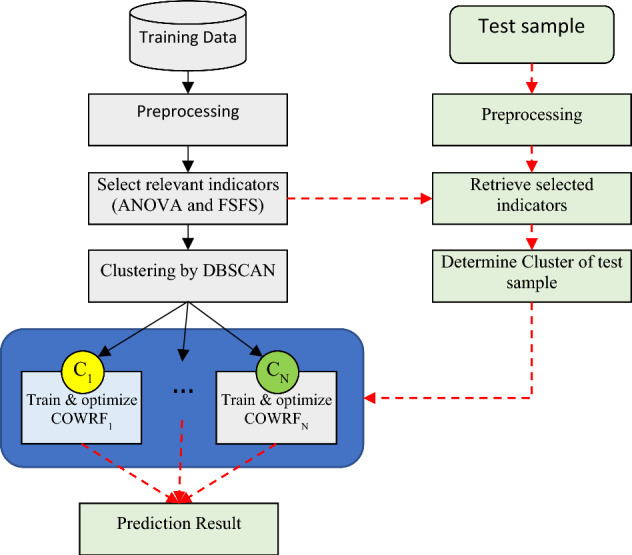
Steps of flight delay prediction in the proposed method.

The mechanism of the proposed method is illustrated in Fig. 1 as a diagram. In this diagram, the stages related to the training and testing phases of the proposed model are separated. To clarify the processes, the training phases are connected through solid black lines, and the testing stages (prediction of delays in new samples) are represented by dashed red lines. The training phase of the proposed method starts with data preprocessing. Then, the indicators of the training samples are ranked based on ANOVA, and the most relevant indicators associated with flight delays are identified using the FSFS algorithm. In the next step, the training samples are clustered based on the selected indicators. The clustering process is performed using the DBSCAN algorithm, which helps identify noise and disregard it in the training samples. By doing so, the training samples are divided into N clusters. In the next step, the samples from each cluster are used to train the proposed prediction model. This model utilizes COA to optimize the tree components and adjust the weight values for these components. The obtained learning models for each cluster are then used to predict delays in new samples.

To predict delays in new samples, the preprocess operations are first applied on the input record, and then, the selected indicators during the training phase are extracted from the input record. Then, the probability of belonging of the test sample to each cluster (C_1_ to C_N_) is calculated, and the sample is assigned to one of the existing clusters. Once the cluster of the test sample is determined, the corresponding COWRF model for that cluster is used to predict the delay. Each COWRF model utilizes a weighted voting strategy among the tree components to determine the prediction result.

#### Preprocessing and selection of relevant indicators

The proposed approach starts with preprocessing and selecting indicators relevant to flight delays. The preprocessing operation starts with handling missing values. If there is a missing value for a continuous indicator, it is replaced with the mean value of that indicator in the training samples. Also, any missing value in categorical indicators is replaced with 0.

After handling missing values, each database indicator is mapped to the range [0, 1] using the following equation^25^:

1 $${N}_{I}=\frac{I-m{in}_{I}}{ma{x}_{I}-mi{n}_{I}}.$$

In the above equation, *I* represents the vector of values for each indicator, and *min*_*I*_ and *max*_*I*_ represent the minimum and maximum possible values for that indicator, respectively. After normalizing the indicators, the process of selecting indicators relevant to flight delays is performed. The prerequisite for this task is determining the importance of each indicator based on its values. For this purpose, the F-score in one-way ANOVA can be used. One-way ANOVA is a suitable method for investigating the effect of each independent variable (input indicators) on the dependent variable (flight delay)^26^. In the proposed method, this analysis is performed on each input indicator to determine the significance level of its effect on flight delay in the form of an F-score. The F-score for each indicator indicates the ratio of between-group variations to within-group variations. If $F>1$, it means that the between-group variations are greater than the within-group variations, indicating that the effect of that indicator on the dependent variable could be beyond random situations. Furthermore, as the F-value increases, the effect becomes more evident. Based on this, in the proposed method, after calculating the F-score for each input indicator, they are ranked in decreasing order by the obtained F-score to rank them according to their importance.

After determining the importance of input indicators, it is necessary to determine the appropriate number of indicators to achieve the highest prediction accuracy. In the proposed method, the FSFS algorithm^27^ is used to fulfill this objective. The FSFS algorithm is a feature selection strategy based on ranked features, which can determine the appropriate number of features for predicting flight delays. In this regard, the learning model is first trained based on the top-ranked two indicators, and the validation error is calculated based on these indicators. In the next step, the third-ranked indicator is added too, and the training and validation error calculation processes are performed, respectively. This process is repeated, and after adding each new indicator, the obtained validation error is compared with the previous state. If adding a new indicator leads to an increase in the validation error, the feature selection procedure is ended, and indicators having minimum validation error will be selected. This selected set is used as the input for the second step of the proposed method.

#### DBSCAN-based clustering

After preprocessing and selecting relevant indicators, the training samples are clustered using the DBSCAN algorithm. This algorithm performs data point clustering based on their density. Accordingly, data points that are close to each other and form a region with high density are considered as a cluster. On the other hand, points that are far from dense regions are identified as outliers^28^. In proposed method the clustering algorithm is applied on all training samples. Each cluster contains a set a training samples which are used to train a separate learning model. Using the clustering strategy for flight data through this algorithm has several advantages. Firstly, the DBSCAN algorithm has the ability to distinguish outlier samples from data points, and employing this strategy can prevent the inclusion of samples that may disrupt the prediction process. Secondly, the use of clustering strategy leads to simplifying the problem by breaking it down into several subproblems with lower complexity. By utilizing the sub-problems generated by the structure of clusters, the prediction process can be performed more accurately. In this case, each learning model is trained based on the controlled set of data in each cluster, which strengthens the model’s ability to learn patterns specific to each cluster. Additionally, clustering the data reduces the training time of the learning models, which is crucial for processing large-scale flight data in real-world applications. These reasons justify the use of the clustering strategy for the samples in the proposed method. However, it should be noted that the clustering algorithm should be capable of processing big data.

The original DBSCAN algorithm has a computational complexity of $O\left({N}^{2}\right),$ which is not suitable for large-scale data processing applications. Therefore, the proposed method uses an enhanced version of the DBSCAN model for clustering the samples. This enhanced algorithm improves the performance of DBSCAN by sampling from the data points and initializing the clusters.

Similar to the original DBSCAN algorithm, this enhanced algorithm utilizes two threshold parameters: the neighborhood threshold ϵ and the minimum number of points P. It is worth mentioning that in this algorithm, the neighborhood set of each data point x, which has a distance less than the ϵ threshold, is denoted as ${N}_{x}$, and the set of data points that have a distance less than $\frac{\epsilon }{2}$ with it is denoted as $N{C}_{x}$. The enhanced DBSCAN algorithm employs three sets, C, M, and I, to organize the cluster structure. These sets represent the core points of clusters, the margin points, and the cluster labels, respectively. The algorithm starts by initializing sets C, M, and I as empty. Then an iterative mechanism is used for assigning a cluster id to each unlabeled sample. During each iteration, the number of neighbors within a distance less than ϵ from data point are determined as $\left|{N}_{x}\right|$. Data points with neighbors larger than threshold P are considered as core points and their unlabeled neighbors are labeled according to their new core. On the other hand, data points with neighbors smaller than threshold P are considered as margin point; while samples without neighbors are marked as noise. After processing all data points, each margin point is assigned with a label using majority of cluster id in its neighborhood. The pseudo code the enhanced DBSCAN is presented in Algorithm 1.

**Algorithm 1:**
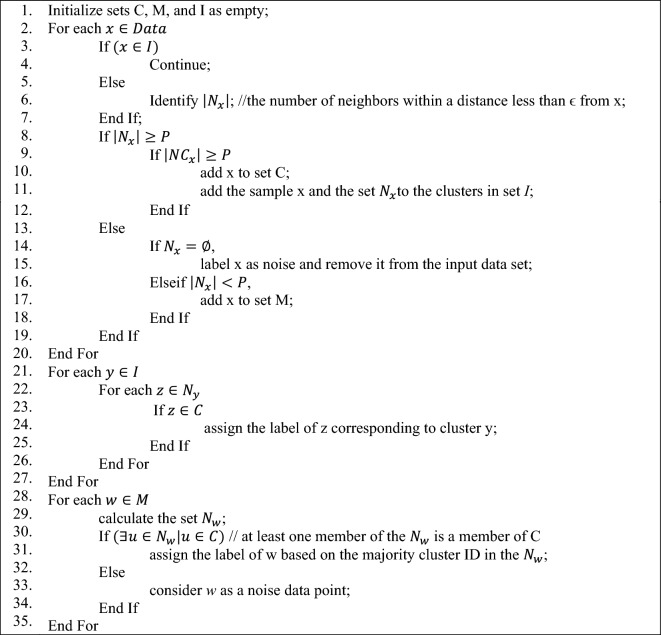
Enhanced DBSCAN.

By executing the above steps, the training samples will be assigned to N clusters. Additionally, the identified training samples as noise will be discarded during the training process. In the next step, each cluster will be assigned a learning model to train the model based on the training samples of that cluster. In this research, the parameters P and ϵ are set empirically to 15 and 0.1, respectively.

#### Predicting delays based on COWRF models

Finally, optimized random forest models by COA are used for training based on the samples of each cluster. To do so, a learning model is assigned to each cluster formed in the previous step. In this study, the proposed learning model for predicting flight delays is called COWRF. In this model, a random forest is initially used to build the base learning model. Then, COA is used for optimization and configuration of the base model at the local and global levels:

1. *Optimizing configuration of each tree model* At this level, COA is used to modify each tree by tuning its split points. Based on these parameters, each decision tree component of the base random forest model is configured. The goal of this step is to minimize the local error of each decision tree component in identifying flight delay patterns.
2. *Determining the effectiveness of each tree model on the detection outcome through assigning weight values* Since the decision tree components in random forest models have different accuracies, the output value of each component can be different from others. To address this issue, the output value of each decision tree component in COWRF is determined using a specified weight value. In this case, accurate decision tree components will have a higher weight, while components with higher errors will have a lower weight. Finally, the output of the COWRF model is determined through weighted voting among these components.

The continuation of this section explains how the COWRF model is optimized at these two levels.

##### Optimization of each tree model configuration

**Figure 2:**
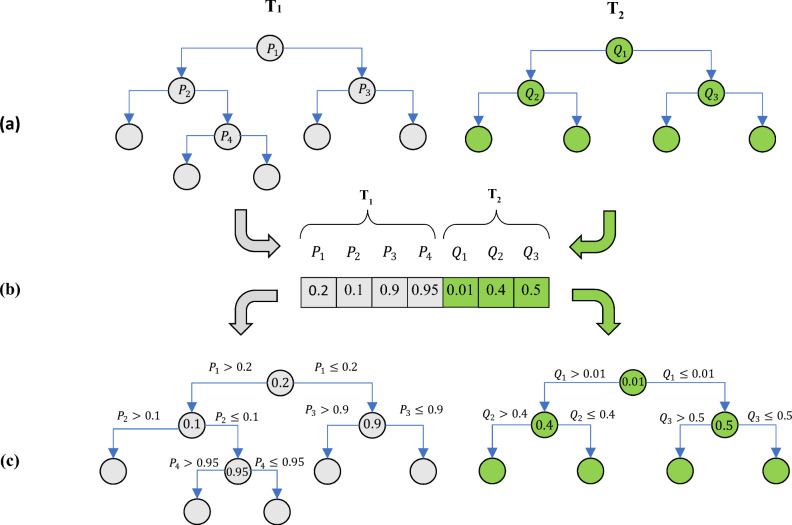
An example of the solution vector and its application on COWRF in optimizing the splitting points (**a**) the initial RF model, (**b**) an example of solution vector in COA, (**c**) the result of applying solution vector on initial RF model.

The algorithms for constructing conventional decision trees utilize greedy strategies to expand the tree structure. In this approach, an appropriate splitting point at the root node is greedily searched to generate two child nodes branching from the root. Then, the process of determining splitting points for each child node is performed in a similar manner, and this process is repeated until the decision tree structure is complete. It is evident that the splitting points formed in greedy algorithms may not lead to an optimal decision tree. Therefore, revisiting the determined values in decision nodes can contribute to achieving an effective decision tree performance. In the proposed method, this task is accomplished using COA. Adjusting the decision points in the components of the COWRF tree can be done separately or collectively. In the first case, each tree component is optimized individually by COA, and in the second case, all decision points in all trees are simultaneously optimized by COA. In this research, the second case is utilized. Furthermore, the structure of the solution vector and the fitness evaluation function in the optimization problem are described, followed by explaining the computational steps of COA for solving this problem. To optimize the splitting points in each component of the COWRF tree, each splitting point is considered as an optimization variable. In this case, each solution vector in COA has a length equal to the number of decision points in all COWRF trees. Since all input indicators are mapped to the interval $[\mathrm{0,1}]$ in the preprocessing step, each optimization variable (splitting point) is represented as a real number within this range. Figure 2 illustrates an example of the structure of the solution vector and its utilization in the problem. In this figure, a COWRF model consists of two hypothetical trees, T_1_ and T_2_, which have 4 and 3 decision nodes, respectively. In this case, according to Fig. 2b, each solution vector has a length of 7, where the first 4 elements specify the split points of decision nodes P_1_ to P_4_ in T_1_, and the next 3 elements correspond to the split points of decision nodes Q_1_ to Q_3_ in T_2_. The process of applying the determined decision points in the COA solution vector on the components of the COWRF tree is illustrated in Fig. 2c.

After setting the splitting points for each component of the tree based on the solution vector, training samples are applied to each configured tree, and the output labels for the input samples are determined. Then, the fitness of the solution vector is evaluated by comparing the output labels of these models with the actual labels:

2 $$fitnes{s}_{local}=\frac{1}{T}\sum_{i=1}^{T}\frac{{e}_{i}}{N},$$

where T represents the number of tree components in the COWRF model, which is considered as 25 in this research. Additionally, N denotes the number of training samples, and ${e}_{i}$ indicates the number of errors made by tree component *i* in labeling the training samples. In other words, in this phase, the COA utilizes the average error rate of tree components as a fitness measure to evaluate the fitness of each solution vector. The COA algorithm starts with randomly generating an initial population consisting of p packs, each containing c coyotes. This algorithm iterates the search mechanism until one of the stop criteria are met. During each iteration, first the coyote with the minimum fitness in each pack is considered as alpha. Then, the social attraction of the current group is calculated based on the following equation^29^:

3 $$cul{t}_{j}^{p,t}=\left\{\begin{array}{l}{O}_{\frac{c+1}{2},j}^{p,t}, \quad if \, c \, is \, odd\\ \frac{{O}_{\frac{c}{2},j}^{p,t}+{O}_{\frac{c}{2}+1,j}^{p,t}}{2} \quad else\end{array}\right..$$

In the mentioned context, ${Q}^{p,t}$ represents the social rank of coyotes in pack p for iteration t.

Then, for each coyote such as c in each pack, the social position of the coyote is updated as follows^29^:

4 $$newSo{c}_{c}^{p,t}=so{c}_{c}^{p,t}+{r}_{1}\times {\delta }_{1}+{r}_{2}\times {\delta }_{2}.$$

In the above equation, r_1_ and r_2_ represent the weight coefficients of the alpha coyote and the current pack, respectively. Additionally, δ_1_ and δ_2_ represent the impact rates of the alpha coyote and the current pack, which are calculated based on the difference between the position of the alpha coyote and the pack position. Then the social position of the coyote is updated based on its fitness changes, and birth and death operators are applied to each pack. In the end of each iteration, the probability of transferring the coyote to another pack is calculated and the age of coyotes are updated. The pseudo code of COA has been presented in Algorithm 2.

**Algorithm 2:**
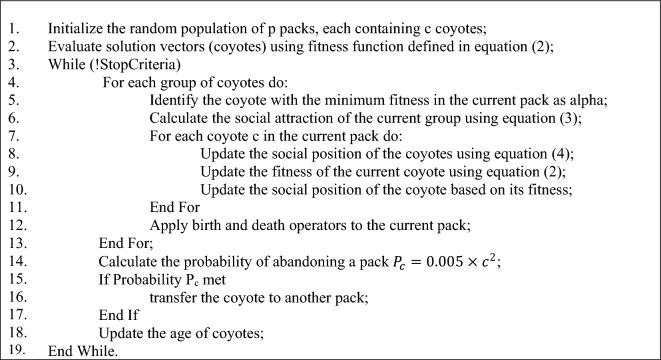
Coyote Optimization Algorithm.

After executing the aforementioned optimization steps, the solution vector with the minimum fitness (lowest average training error) is applied to the tree components.

##### Weight assignment in COWRF

In order to predict flight delays in the COWRF model, the input sample is applied to each tree component, and the final output of COWRF is determined by majority voting among the outputs generated by these components. However, it should be noted that each tree model may have different performance compared to others. Therefore, the output value of each tree model in COWRF may vary from others. Consequently, the negative effect of models with high errors can lead to errors in the proposed prediction system. To address this issue, the weighted strategy for tree components is employed in COWRF. To achieve this, the influence of each tree’s output in the COWRF structure is determined using a specific weight value, and the output of the proposed model is determined using the weighted voting strategy. COA is also utilized in this phase to assign weight values to each tree component. In this case, COA tries to improve the overall accuracy of the prediction system by assigning lower weights to trees with high errors and increasing the weights of more accurate trees. The optimization steps for weight values in this phase are similar to the previous phase, but a different structure for describing the solution vector and evaluating fitness is used. Therefore, in this section, we will focus on explaining the structure of the solution vector and the fitness function.

The objective of COA in this phase is to allocate a weight value to each tree component in COWRF. Therefore, the number of optimization variables is equal to the number of tree components in COWRF. Each optimization variable determines the weight value corresponding to a tree, represented as an integer in the range [0,10]. With these explanations, for a COWRF model consisting of N trees, the solution vector is described as an integer vector with a length of N. The assigned weight value to each tree component determines the number of times its output is counted in the voting process. For example, if a tree has a weight value of 3, its output will be counted 3 times for each sample in the voting process. Similarly, assigning a weight of 0 to a tree indicates its exclusion from the voting process.

To evaluate the fitness of each solution vector in this phase, the validation samples are applied to the tree components in COWRF, and the output of each model corresponding to its weight value is repeated. Then, the voting process is performed based on the repeated output vectors of the models to determine the COWRF’s prediction result for each validation sample. Finally, the fitness evaluation is conducted by the validation error as follows:

5 $$fitnes{s}_{global}=\frac{e}{N},$$

where e refers to the size of the validation set for which the COWRF output contradicts the ground truth labels, and N denotes the total number of validation samples. Using this fitness measure, the combination with the minimum validation error can be determined for the prediction model. Ultimately, the COWRF model with the optimal weight combination is utilized for predicting delays in new flights.

## Results and discussion

To implement and evaluate the proposed method, MATLAB 2020a software was used. The experiments in this research were conducted based on the 50-fold Cross-Validation (CV) mechanism. Considering the size of the dataset, and the issue of class imbalance, 50-fold CV was considered as an appropriate and effective strategy to evaluate the proposed model. By repeatedly dividing the data into smaller folds and training the model on different subsets, we can obtain more reliable and unbiased performance estimates. This approach helps to reduce the impact of random noise and ensures that the model’s performance is not overly influenced by the distribution of data points in a particular fold. Therefore, during the 50-fold CV, the training and testing processes of the model were repeated 50 times, and in each iteration, 98% of the samples were used for training the model, while the remaining 2% were used for evaluating its performance. It should be noted that this principle of data partitioning has been used for the samples of each class in each fold of CV. In other words, the training samples in each fold of CV include 98% of the samples of each class; while the remaining 2% of the samples of each class were used for model testing in that fold. Since new test samples are used in each fold, after completing the evaluation, all database samples will be used as test samples. In each iteration, the performance of the proposed method was evaluated based on accuracy, precision, recall, and F-measure metrics, and the average results are presented in this section.

The accuracy metric represents the ratio of test samples (in all target classes) that are classified correctly. On the other hand, the precision metric indicates the proportion of correctly classified samples in each class, i.e., on-time, low delay, and high delay. Additionally, the recall metric shows the relative ratio of correctly identified samples in each target class. It should be noted that to calculate precision and recall metrics, the problem should be considered as a binary classification problem (positive and negative classes). Accordingly, these metrics were calculated separately for each target class. In this case, for each class, it was considered as the positive class, and the other classes were considered as negative. These metrics are calculated based on the following equations^30^:

6 $$Precision=\frac{TP}{TP+FP},$$

7 $$Recall=\frac{TP}{TP+FN},$$

8 $$FMeasure=\frac{2\cdot Recall\cdot Precision}{Recall+Precision}.$$

To examine the specific impact of each employed technique on the performance of the proposed method, the results obtained from this strategy were compared with the following scenarios:

- *All Features* In this scenario, flight delay prediction is performed based on all features (Listed in Table 1). In other words, the process of selecting relevant indicators is disregarded, and the performance of the COWRF model is evaluated based on all features.
- *Without DBSCAN* In this scenario, the clustering process of the samples by the DBSCAN algorithm is disregarded, and only a COWRF model is used to classify all samples.
- *Conventional RF* In this scenario, the proposed COWRF model is replaced with a conventional random forest model, and flight delay prediction is performed based on this model.

The goal of examining the first scenario is to examine the effect of the feature selection strategy in improving the performance of the flight delay prediction system. Based on the second comparison scenario, the impact of clustering the samples on the accuracy of the proposed model can be demonstrated. The third comparison scenario indicates the effect of optimizing the tree components and their weighting by COA on increasing prediction accuracy. Additionally, the results of the presented approach were compared with the DFCNN model in Ref.^13^, the CCRF model in Ref.^14^, the CNN-LSTM mode in Ref.^18^ and GHOA-RF model in Ref.^20^. Same training and test sets were used for all of these methods.

**Figure 3:**
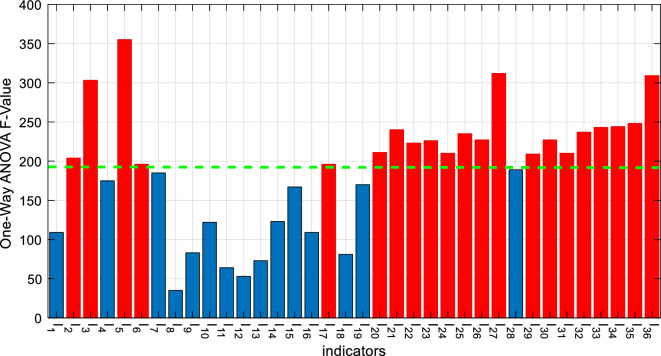
F-scores calculated based on ANOVA for input features.

According to the procedure described in “Preprocessing and selection of relevant indicators” section, the identification of features related to flight delay was performed using ANOVA and the FSFS strategy. First, the F-score of the features was evaluated based on the ANOVA test. Figure 3 shows the calculated F-scores for the input features. These results indicate that weather features, airport congestion characteristics, and some flight-related attributes such as aircraft type and route length are more important. After sorting the features based on their F-scores and applying the FSFS strategy for feature selection, it was found that the highest prediction accuracy can be achieved using 21 selected features. These features are distinguished from other features by a green dashed line in Fig. 3. Therefore, the presented results in this section are based on these 21 selected features.

**Figure 4:**
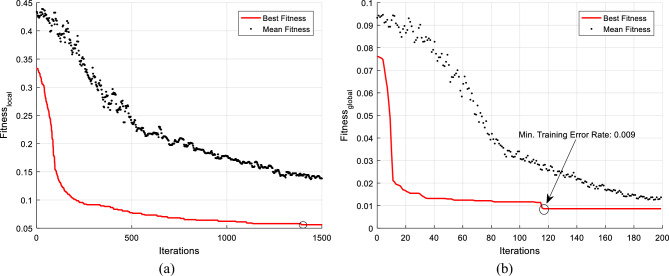
Variations of the best fitness during various cycles for (**a**) tree configuration optimization phase and (**b**) tree weighting phase in the COWRF model.

In the proposed method, COA was used for optimizing the COWRF model. This optimization was performed in two phases. In the first phase, the process of tuning the decision points of the trees was performed, and then, the effect of each tree component on the output of COWRF was determined using a specified weight value. It should be noted that in the first phase, the parameters of the number of packs, the number of coyotes in each pack, and the number of COA iterations were set to 50, 10, and 1500, respectively. Additionally, in the second phase, these parameters were set to 10, 10, and 200, respectively. Figure 4 shows the variations of the fitness in the best solution found during various iterations for each of these optimization steps. According to Fig. 4, the COA algorithm can minimize the fitness value through efficient search in the problem space in both phases. According to these graphs, the average population fitness in both phases also showed a decreasing trend towards the best discovered fitness. The optimization of the COWRF model at the local level (configuring each tree) and the global level (weighting the models) was effective in further reducing the training error. According to Fig. 4a, the optimization of each tree’s configuration at the first level was able to reduce the average training error of the tree components in COWRF to about 5%. After that, weighting the learned models at the second level led to further reduction in the error, resulting in a weighted model with a training error of less than 1%. These results confirm the effectiveness of the proposed two-level optimization technique.

**Figure 5:**
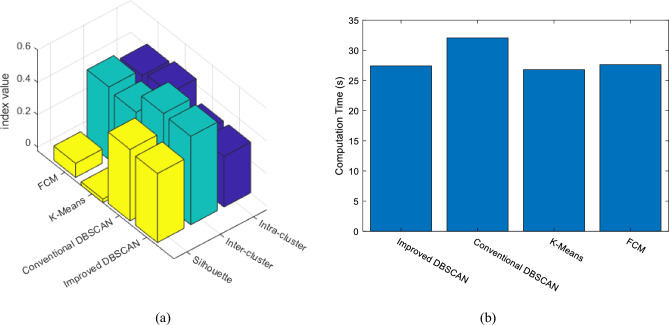
The results of evaluating the performance of clustering algorithms in terms of (**a**) clustering quality, and (**b**) processing time.

Before examining the performance of the proposed method in predicting flight delay, it is useful to evaluate the performance of the improved DBSCAN algorithm in clustering samples. For this purpose, the clustering quality of database samples based on this algorithm has been compared with other clustering algorithms. In this case, the quality of the formed clusters has been evaluated based on the intra-cluster distance, inter-cluster distance and silhouette index. Also, the efficiency of the improved DBSCAN algorithm has been compared with other clustering methods in terms of processing time. This experiment was performed on a personal computer with an Intel Core i7 processor with a frequency of 3.2 GHz and 16 GB of memory. The results of this experiment are presented in Fig. 5.

Figure 5a, compares different clustering algorithms using intra-cluster, inter-cluster and silhouette index. Also, Fig. 5b compares these methods in terms of processing time. The intra-cluster distance is calculated as the average distance between each pair of data points of the same cluster. Also, the inter-cluster distance represents the minimum average distance between the points of each cluster and the points of other clusters. Finally, the silhouette index is calculated as follows^31^:

9 $$S=\frac{b-a}{{\text{max}}(a,b)}.$$

In the above relationship, *a* and *b* represent the intra-cluster and inter-cluster distances, respectively. It should be noted that distances have been calculated based on normalized features (Eq. 1). As the results presented in Fig. 5 show, the improved DBSCAN algorithm can organize the data into clusters with less inter-cluster and more intra-cluster distances, which results in increasing the silhouette index and achieving clustered structures with higher quality than clusters. On the other hand, compared to the conventional DBSCAN algorithm, the improved model can perform clustering in a shorter period. Based on the results, the improved DBSCAN algorithm, while having the qualitative advantages of the conventional DBSCAN algorithm, can compete with faster algorithms such as K-Means in terms of processing time. These results show that the clustering technique used in the presented approach can be more suitable for big data processing applications.

**Figure 6:**
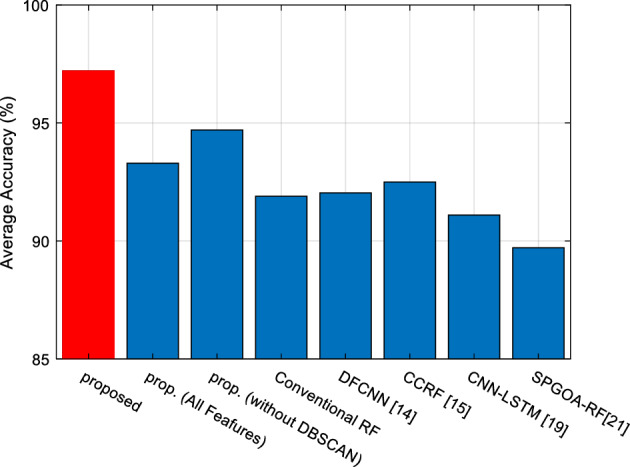
Mean accuracy in flight delay prediction.

Figure 6, represents the mean accuracy in flight delay prediction after 50-folds of cross-validation are presented. The examination of the accuracy values of different scenarios shows that the proposed method is capable of achieving more accurate flight delay prediction in the database samples. The average accuracy of the proposed method is 97.2%, which is at least 2.49% higher compared to the closest scenario. Moreover, the proposed COWRF model outperforms DFCNN, CCRF, CNN-LSTM, and GHOA-RF methods in terms of accuracy, even without using the clustering and feature selection strategy, and it can achieve flight delay prediction more accurately than these two methods.

By comparing the different scenarios in Fig. 6, valuable insights can be extracted regarding the performance of the proposed method. For instance, if the process of selecting relevant features is disregarded, the prediction accuracy would be 93.29%. This means that the process of selecting relevant features can increase the accuracy by an average of 3.91%. It is likely that with the adoption of more efficient feature selection strategies, this difference can be further increased. On the other hand, if only a single COWRF model is used for flight delay prediction (eliminating the clustering step in the proposed method), the prediction accuracy would decrease to 94.70%. This indicates that the clustering strategy and problem decomposition played an effective role in reducing complexity. Employing a separate COWRF model for each cluster allows the models to learn patterns in each cluster more effectively, resulting in a 2.5% increase in accuracy. Additionally, the clustering strategy leads to faster detection in the proposed model, as it can reduce the processing time for each sample by an average of 39.17%. Furthermore, the COWRF model outperforms the conventional random forest model by 5.3% in terms of accuracy. These results demonstrate that even without the COWRF model and using prediction based on the baseline random forest, the proposed method achieves a slight advantage over the CCRF model^14^. This advantage confirms the effectiveness of the feature selection and clustering strategies in reducing the complexity of the problem.

**Figure 7:**
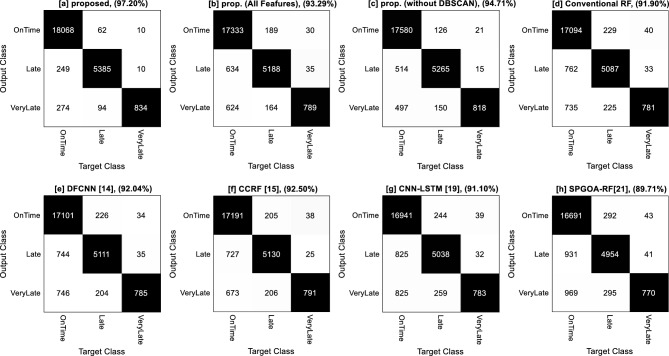
Confusion matrices of different methods in flight delay prediction.

Figure 7, presents the confusion matrices resulting from flight delay prediction by different methods. In each matrix, the rows indicate the prediction of flight delay by different methods, and the columns represent the actual distribution of samples in the target categories. For example, Fig. 7a demonstrates that the presented approach correctly predicted 18,068 out of 18,591 on-time flights (sum of values in the first column) and incorrectly classified 249 on-time flights as delayed flights. Also, 274 on-time flights were incorrectly classified as flights with high delays. Furthermore, there were 5541 flights with low delay, and the presented approach correctly identified delay in 5385 flights and misclassified 156 samples in other categories. The interpretation of the confusion matrix can be performed similarly for other methods.

Examining the performance of different methods separately for each category in the confusion matrices reveals that, generally, the detection of flights without delay is more accurate than flights with delay. One reason for this is the larger number of samples in the category, allowing the learning models to more accurately identify the underlying patterns in the features of on-time flights. Another reason is the high similarity between the features in the two categories of flights with low and high delay, which, coupled with the small number of samples, leads to the models' confusion in distinguishing between these two categories.

**Figure 8:**
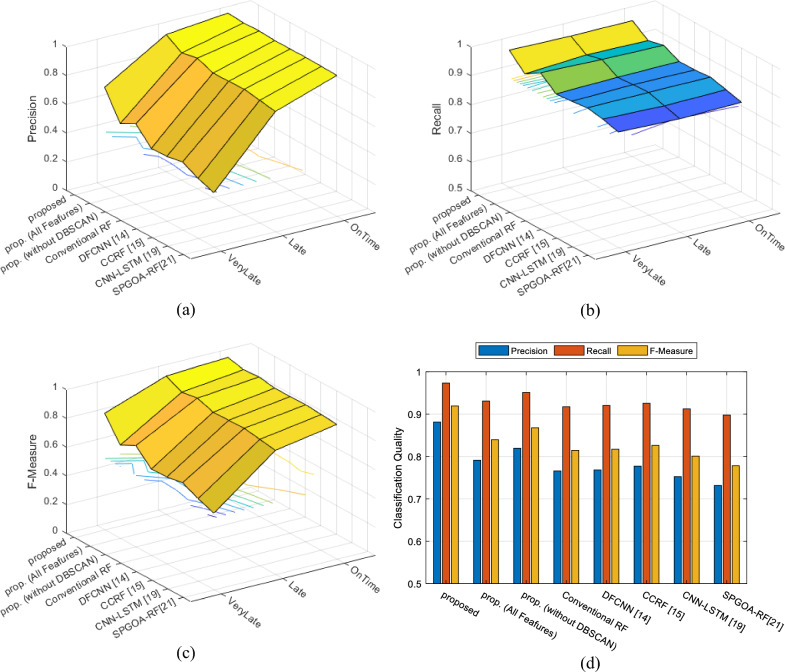
Performance of different methods in terms of (**a**) precision, (**b**) recall, and (**c**) F-measure for each class and (**d**) the average values of these metrics.

By comparing the confusion matrices, two points can be inferred. Firstly, the presented approach, in addition to being more accurate, also performs more accurately in categorizing samples in each category, indicating its superiority in terms of accuracy for each category. Secondly, the proposed method is capable of correctly identifying a higher rate of samples in each category, resulting in higher recall compared to the compared scenarios. This claim can be confirmed through Fig. 8. In this figure, the performance of different methods in terms of accuracy, recall, and F-measure for each category (Fig. 8a–c) and as an average (Fig. 8d) is presented. In Fig. 8a–c, the x-axis represents different methods, and the y-axis represents each target class. The values in these graphs represent the quality measures of classification for each target class. This figure clearly demonstrates that the proposed method is capable of improving the quality measures of classification in each target category. Furthermore, the average values presented in Fig. 8d clearly indicate the superiority of the presented approach. This superiority can be the results of using the strategies employed in feature selection, data clustering, and classification processes.

**Figure 9:**
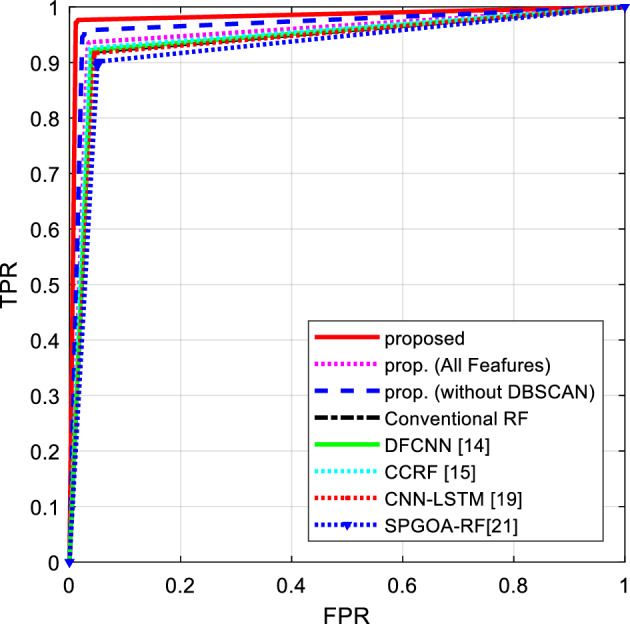
ROC curves resulting from flight delay prediction by different algorithms.

Figure 9, presents the ROC curves resulting from flight delay prediction by different methods. It should be noted that for drawing the ROC curve for each method, the Late and VeryLate categories were merged and labeled as the positive class. Thus, the ROC curve can effectively describe the performance of each method in predicting delay for each flight.

As shown in Fig. 9, the presented method achieved a lower false positive rate and a higher true positive rate in predicting flight delays. The reduction in the false positive rate indicates that this approach misclassified fewer on-time flights as delayed. On the other hand, the increase in the true positive rate demonstrates the favorable efficiency of the presented approach in predicting the presence of delays in flights. Table 2, represents a summary of the results obtained from these experiments.

**Table 2:** Summary of the results obtained from the experiments.

Method	Accuracy	F-measure	Recall	Precision
Proposed	97.2024	0.9193	0.9734	0.8813
Proposed (all features)	93.2922	0.8395	0.9308	0.7912
Proposed (without DBSCAN)	94.7050	0.8675	0.9512	0.8196
Conventional RF	91.8995	0.8145	0.9174	0.7660
DFCNN^13^	92.0395	0.8173	0.9205	0.7684
CCRF^14^	92.4998	0.8265	0.9256	0.7773
CNN-LSTM^18^	91.0990	0.8009	0.9124	0.7525
GHOA-RF^20^	89.7102	0.7782	0.8978	0.7316

The analysis of the results obtained from the conducted experiments indicates that the proposed method is an efficient and accurate strategy for predicting flight delays in both domestic and international flights, and it can serve as a useful tool in real-world scenarios.

## Conclusion

Accurate prediction of flight delays is an important and challenging problem in the aviation industry, and achieving it can improve the efficiency of flight-related processes. However, challenges such as low accuracy and the lack of compatibility of machine learning models with large-scale flight data have hindered their practical use. In this research, the focus was on addressing these challenges and presenting a novel method for accurate detection of flight delays. To achieve this, a combination of ANOVA and FSFS strategies was initially used to determine the most relevant features associated with flight delays. The analysis revealed that besides weather-related features, certain flight characteristics such as aircraft type, flight type, and congestion-related attributes, as well as departure delay history, had the greatest impact on the occurrence of delays. The feature selection strategy was found to effectively increase the prediction accuracy by at least 3.91%. In this study, a clustering strategy was also employed to decompose the problem and reduce its complexity. The samples were clustered into sets of similar categories using the DBSCAN algorithm, and a separate learning model was utilized for predicting delays in each category. According to the findings, the clustering technique improved the speed and accuracy of delay prediction by 39.17% and 2.49%, respectively. Furthermore, the employed learning model for each cluster was a random forest, with the adjustment processes of decision node splits and weight determination for each tree component performed using COA. This new ensemble model could enhance the prediction accuracy by 5.3% compared to the conventional random forest model. The evaluation of the proposed method based on real flight data from JFK airport demonstrated that it achieved an average prediction accuracy of 97.2%, indicating a 4.7% improvement compared to previous efforts.

Although the significance of feature selection in improving flight delay prediction was demonstrated in this research, employing more efficient feature selection techniques could lead to even better results. Therefore, this aspect can be further explored in future studies. Moreover, in prediction applications, a wide range of features may be associated with uncertainties. Hence, by integrating the proposed model with a fuzzy model, a more accurate description of flight features can be achieved, aiming to improve the model’s flexibility.

## Data Availability

All data generated or analysed during this study are included in this published article.
